# Decade effects in mental addition

**DOI:** 10.1098/rstb.2024.0220

**Published:** 2025-10-20

**Authors:** Hansjörg Neth, Stephen J. Payne

**Affiliations:** ^1^ Department of Psychology, University of Konstanz, Konstanz, Germany; ^2^ Department of Computer Science, University of Bath, Bath, UK

**Keywords:** mental arithmetic, base notation, decimal system, addition strategies, representational effects

## Abstract

We examine representational effects of Western numerals on mental arithmetic. An analysis of mental addition tasks using a base-10 place-value notation yields a taxonomy of addition types that is anchored in the notion of complements (i.e. additions with round sums). Two experimental studies use a paradigm of serial addition that presents lists of numbers to adult participants, who mentally represent all intermediate steps. In study 1, participants add sequences of single-digit addends in a self-paced fashion. Study 2 extends this paradigm by simultaneously presenting two addends, thus allowing for a modicum of strategic choice. Both studies vary the number of complements within the lists and measure addition accuracy and latency. Beyond decade and carry effects, our results show that lists containing or enabling complements are easier to add. Addition latencies jointly depend on addition type and problem size. When adders have some discretion about the order of choosing addends, they adaptively exploit the difficulty of addition types by tailoring their sequences to decade boundaries. One motivation for seeking complements lies in enabling subsequent post-complements. Reflecting on the dynamic interplay between numeric representations, strategic choices and cognitive adaptations, we discuss implications for psychological explanations, technology and design.

This article is part of the theme issue ‘A solid base for scaling up: the structure of numeration systems’.

## 1. Introduction

The most important part of the Analytical Engine was undoubtedly the mechanical method of carrying the tens. (…) The difficulty did not consist so much in the more or less complexity of the contrivance as in the reduction of the time required to effect the carriage. (…) nothing but teaching the Engine to foresee and then to act upon that foresight could ever lead me to the object I desired…

Charles S. Babbage (1864), *Passages from the Life of a Philosopher,* p. 114

Babbage’s conundrum has nothing to do with the mathematical aspects of number or arithmetic, and everything to do with properties specific to the base-10 place-value notation in which numbers are ordinarily represented [1]. What are the effects of adopting the Indo-Arabic decimal system—also known as the Western numerals [2]—on the processes of mental arithmetic? The very notion of mental arithmetic suggests that any effects are limited to the translation between input/output formats and some mentalese in which the operations are performed [3]. Contrary to this intuition, we will argue that our notational system affects the relative ease with which elementary additions are mentally computed.

In particular, we develop, empirically, the following argument. First, the use of the Western numerals leads to a variety of problem difficulty effects, in which some elementary operations are easier than others depending on their parameters. Second, that people are in some sense aware of this gradient of difficulty, in so far as they adaptively adopt strategies for multi-operation processing that use easier rather than more difficult component steps.

In developing this argument, we contribute to three important traditions in work on mental arithmetic. First, there is a long tradition of representational effects (see [4–6] for reviews). Much of this work has focused on representations of numeric magnitude and on notational differences, such as comparing arithmetic procedures in Roman versus Western numerals [7–10]. We will instead focus on the implications of using Western numerals for the relative difficulty of mental addition tasks. Second, there is work on problem-difficulty and especially problem-size effects [11–15]. This tradition has focused on the relative ease of primitive operations (e.g. encoding, counting and computing quantities or retrieving arithmetic facts) that are often assumed to be independent of the notational system being used. Third, there is work on arithmetic strategies, how they are discovered, develop over time and the extent to which they are adaptive [16–19]. This work has mostly been concerned with algorithms for solving various arithmetic tasks (e.g. of addition, subtraction multiplication), whereas we target strategies for combining primitive operations in multi-step addition. In the following, we will briefly sketch some issues raised by these traditions that directly motivate our research.

### (a) Background

Research on *representational effects* in numerical cognition has traditionally focused on number size and distance effects [20]. When comparing two numbers, the fact that performance is better when the numbers are smaller (2 versus 4 is easier than 6 versus 8) or their difference is larger (2 versus 8 is easier than 4 versus 6) challenges simple discrete theories of number representation (see [21,22] for recent accounts). Studying the influence of notational systems on the ease of mental operations, theoretical comparisons of Roman with Western numerals have pointed out that different notations enforce specific computational constraints and selectively facilitate different subprocesses [7–10]. Related empirical work has shown that notational effects can be measured for internal operations and interactive read-write processes. In a verification paradigm displayed in a mixture of Roman and Western numerals, the reaction times of very simple addition problems (with sums below 10) were influenced by notation [23]. This suggests that distinct mental operations are applied to different numerals, so that the notation exerts an effect on internal transformation processes [24]. Research on linguistic effects on numerical cognition has shown that arithmetic problems are easier when number concepts map onto the structure of the task. For instance, higher base transparency in number names is positively related to the counting skills of pre-schoolers speaking English versus Mandarin [25]. Similarly, Basque speakers expressing the number word for 35 as ‘twenty and fifteen’ are faster to compute 20+15 than 25+10, even when problems are presented and entered as Western numerals [26]. Perhaps the most striking evidence for the impact of representations on mental arithmetic comes from Chinese abacus experts [27]. Not only were the solution times of skilled abacus operators found to be based on the number of corresponding steps, but their errors were accounted for by misplacing beads on their mental devices. Thus, there is ample evidence that the symbolic, linguistic and physical representation of numbers impact key aspects of numerical cognition.

The second, and perhaps most populated area investigates *problem-size effects* [11–15]. A considerable amount of research has shown that tasks involving larger numbers (e.g. 4+5) are solved more slowly than those involving smaller numbers (2+3). Despite their ubiquity, explanations for problem-size effects remain controversial [28]. Interestingly, it has been suggested that problem-size effects might be related to the issue of strategy choice in that they could partly be explained as a methodological consequence of averaging over participants or problems [29,30]. Although our interest in the impact of an external notation on internal arithmetic operations is not based on problem size, we consider it as an alternative explanation.

A third key area of research addresses the *strategies* that are used to perform arithmetic operations [16–18]. This tradition demonstrates that a surprising variety of strategies are habitually used to solve even elementary addition or multiplication tasks [31,32]. For example, when asked to add 3+6 one might retrieve the answer 9 from memory. Alternatively, one might count up 6 from the number 3. Or, rather more efficiently, one might break the left–right order to start with the larger number 6 and count up 3 from it. While a majority of studies has investigated the issue of strategy choice in developmental settings, strategy selection effects have also been shown for competent adults [33,34]. A recurring theme throughout this research is the choice between fact retrieval, some stepwise procedure (like counting) or the use of more complex algorithms [35,36]. Studies on estimation have shown that rounding is widely used as a heuristic for quick approximations by both children and adults [37–39]. When combining rounding with compensation strategies (e.g. solve 48+25 by adding 2 to get 50+25=75, then deduct 2 to obtain 73), arithmetic tasks can be solved flexibly yet efficiently [40,41].

Most pertinent to the present research are decade number and crossing effects in multi-digit number processing [42]. In contexts using Western numerals, decade numbers (e.g. 10, 20) occur with a higher frequency than other numbers of similar magnitude [43], require shorter reading times [44] and facilitate the addition of single-digit numbers [45]. When adding numbers, carry effects occur when the unit sum of all addends reach or exceed decades and require incrementing the decade digit (e.g. 15+6=21). Problems requiring carries are not just slower and more error-prone than those that do not, but the number and value of carries jointly affect performance [46,47].

The present research is motivated by these decade effects and two observations in the strategy choice tradition. LeFevre *et al*. [30] introduced an analysis of addition tasks that is sensitive to the properties of the Western decimal system: when the sum of a pair of digits exceeds 10, adders are more likely to use a counting rather than a fact retrieval strategy, which may partially explain problem-size effects [30]. Furthermore, additions with sums greater than 10 (e.g. 6+7) are frequently *decomposed* into two stages: up to the decade (6+4) and beyond (10+3). Interestingly, even adults often use decomposition as a back-up strategy, particularly when solving problems with larger values [15]. If this strategy is indeed widespread, it makes the interesting prediction that additions that sum to 10—which we will call *complements*—are likely to be the most practised of all additions. In this case, one would predict that sums adding to 10, or more generally, sums exactly reaching a decade boundary will be even easier than smaller sums. One reason why this complement effect would be intriguing is that it could be pitted against the problem-size effect.

### (b) Terminology

To avoid ambiguities in the following sections, we introduce some terminological distinctions. The arithmetic operation of addition in ℕ, commonly signalled by the infix operator ‘+’, is a function of N×N→N. When adding u+a=s, u is called the *augend*, a is called the *addend* and s is called the *sum*. Thus, an augend is the number to be increased by an addition, whereas an addend is the number by which the augend is increased to jointly result in the sum. Our typology builds upon the notational properties of representing numbers in the base-10 place-value system known as the Western numerals [2]. Throughout this article, we assume that numbers are visually represented in this system. In the case of double-digit numbers, we will abbreviate augends as Au (as a shorthand for A⋅10+u) and refer to the (capitalized) A as the *augend decade* and the (lowercase) u as the augend *unit*. If u equals zero, Au denotes a *decade* or *round* number. As our results demonstrate that the accuracy and latency of mentally computing Au+a=s depend on Au or s being round numbers, we report decade effects in mental addition tasks.

## 2. General methodology

In the following, we report two studies that were conducted on the same group of participants. As their tasks and setups are similar, we describe their shared elements before reporting study-specific details and results.

### (a) Task paradigm

The task paradigm employed throughout this article is *serial addition,* rather than the more conventional presentation of many production tasks each requiring a single addition. Participants are presented with lists of single-digit numbers, one or two addends at a time, and have to respond with the final sum upon receiving a cue which signals the termination of the list. In this paradigm, the running total, which is always maintained mentally, is the augend, and any externally displayed digit is an addend of an addition task (the only exceptions being the first presented number of the list, which provides the first augend, and the last mentally represented augend, which also provides the final sum).

We use the serial addition paradigm, rather than presenting many individual addition tasks, because it is naturalistic—as tasks involving the addition of several numbers occur frequently in everyday contexts—and offers several advantages. Theoretically, the fact that only addends are displayed externally renders the roles of addends and augends unambiguous and reduces the impact of available strategies when solving simple addition problems. Methodologically, adding lists of numbers eliminates the need for entering intermediate results and thus allows for more precisely measuring each addition’s latency. Pragmatically, the serial addition paradigm allows presenting more additions in a fixed amount of time, and a wider range of problems, so that no frequent responses get practised or primed.

Despite its advantages, the serial addition paradigm also provides two methodological challenges. With respect to stimulus design, the existence of $9^{n}$ distinct sequences of n digits 1–9 prohibits the presentation of complete sets. Even excluding repetitions would still allow for a vast number of possible stimuli. By contrast, studying only a small set of lists would risk limiting any effects on the particular properties of the chosen stimuli. We address this issue by generating random stimuli sets for every participant. While ensuring that each set meets constraints that control for explanations based on problem size, the fact that every participant receives a unique set of stimuli boosts external validity without compromising on experimental rigour. A second challenge of our paradigm is that adding lists allows for more and a wider variety of errors than individual additions. Beyond making computational mistakes, participants can forget intermediate results, make typos upon entering results, or enter them after a time limit expires. It is possible to make multiple errors on a list, or erroneously add a list more than once, as incorrect trials are repeated at a later time. To maximize measurement validity, we use conservative measures of addition accuracy and latency. For measuring accuracy, we only consider the first presentation of a list. Similarly, we only use correctly added lists for measuring addition latency.

### (b) Participants

Twenty-one undergraduate students of Cardiff University (16 female, 20 majoring in Psychology) participated in the experiment in partial fulfilment of a course requirement. The mean age of the participants was 21.5 years (ranging from 19 to 39 years) and 90% were in their first or second year of study. All participants had normal or corrected-to-normal vision and were able to correctly identify a set of digits on the screen.

### (c) Apparatus

Participants were studied individually in a quiet laboratory room. The experimental software was programmed in Microsoft Visual Basic TM6 and was running on a personal computer (Intel Pentium TM4, 512 MB RAM) with a flat 17″ screen that was viewed from a 60 cm distance. Numeric digits were presented with a height of 1 cm in bold black Arial font and centred within a white rectangular display area extending 30 cm horizontally and 18 cm vertically at a 1024-by-768 screen resolution. The advance through the programme was entirely keyboard-driven. Participants pressed the ‘Enter’ key to proceed and entered their results on a numerical keypad.

All statistical analyses in this article use logistic or linear mixed-effects models that include participants as a random effect to control for individual differences in adding ability. These models are implemented in R and use the lme4 and emmeans packages [48–50]. Raw data and analysis scripts are available at https://osf.io/j4w32/.

### (d) Procedure

After providing informed consent, participants were told that the key performance measure was the time taken to correctly add lists of single-digit numbers and instructed to add ‘as quickly as possible without making errors’. They were aware that the experiment contained two parts and each part would end once they had correctly added a total of 30 lists.

After receiving instructions, participants performed a practice task in which they correctly added four lists. Both during this practice task and throughout the following test phase, progress through a list was entirely paced by the participant. Participants pressed the ‘Enter’ key to advance and present the next number. At the end of a list, which could not precisely be predicted by the participant, a prompt screen appeared instead of a new number, instructing the participant to enter their current running total (using the number keypad) as quickly as possible. A 2 s time limit was placed on participants’ entry of the sum, to prevent participants from remembering clusters of numbers rather than adding them one at a time.

After entering a result, participants received feedback on the correctness of their answer and pressed a key to signal their readiness for the next trial. They were encouraged to pause between lists for as long as they wished, but were asked to proceed as quickly as possible within any given list. A traffic light signal (switching from red to yellow to green, each phase lasting for 800 ms) allowed participants to prepare themselves for the next trial. Both study parts were separated by a short break. When a participant was ready to continue, they were instructed that they were to add another set of 30 lists in study 2 (but now with two addends being displayed, see below).

As it is impossible to know what exactly participants had added when responding incorrectly, lists of erroneous trials were re-presented at the end of the stimulus sequence. Participants were informed that their goal to complete each study was ‘to correctly add 30 randomly generated lists as quickly as possible,’ but were unaware of the repetition of erroneously added lists.

## 3. Study 1

In this study, participants serially added lists of single-digit addends (ranging from 1 to 9). As the current augend was mentally represented, exactly one addend was externally visible at any time.

### (a) Task analysis

The observation that additions crossing a decade boundary are frequently decomposed into two parts [15,30] can be extended into a task analysis of mental addition that is sensitive to the employed notational system. We propose four qualitatively distinct types of addition and make predictions about their speed and accuracy. Table 1 defines and exemplifies the four basic *addition types*, as well as our corresponding notation (see [51] for a similar typology). If the augend unit and the addend of an addition add up to the next decade, we call this a *complement* [=], as the addend complements the augend to a round sum. If the augend of an addition is zero or a round number, we call this a *post-complement* [−], as this type is normally pre­ceded by a complement and merely involves incrementing the unit position by the numerical value of the current addend. Additions with non-round augends, in which the sum either fails to reach or crosses the next decade boundary, are termed *subcomplements* [<] and *super­complements* [>], respectively. By anchoring our typology in the notion of a *complement*, our addition types are characterized by the relation of the augend and sum of an addition to the base (or radix) of the place-value notation used to represent them.

**Table 1.** Classification of *addition types* for adding a single-digit addend a (with a∈{1…9}) to an augend Au (u denoting the augend's unit).

*addition type*		task features		example
name	notation	u	u+a	
*post-complement*	[−]	=0	<10	10+5
*sub-complement*	[<]	>0	<10	15+4
*complement*	[=]	>0	=10	15+5
*supercomplement*	[>]	>0	>10	15+6

### (b) Method

(i) Design

This study employed a fully within-subjects design, with *list type* (no, one or two complements per list) being the main experimentally controlled factor. The second factor *addition type* [−, <, =, >] was randomized within participants.

(ii) Materials

To maximize the variability of lists and additions, each participant received a unique set of 30 lists, which were computer-generated during the experiment. Each list consisted of four to six single-digit numbers from 1 to 9, without any repetitions of adjacent elements. Through this method of generation, materials were controlled at the level of *list type,* but the particular elements within lists (addends, augends) varied randomly within pre-specified constraints. Three *list types* of 10 lists each were distinguished. Lists in set 1 contained *no* complements (e.g. 2, 9, 4, 2, 6, 4), lists in set 2 contained exactly *one* complement (e.g. 2, 1, 4, 3, 8) and lists in set 3 contained exactly *two* complements (e.g. 6, 4, 2, 7, 1). To render the three sets of lists comparable, and to preempt alternative explanations of the hypothesized effects, sets had similar sizes and sums, but those with more complements had fewer elements and lower sums than those with fewer complements (see appendix Ab for details).

(iii) Hypotheses

On the level of adding entire lists of numbers, we expect facilitation effects (with respect to accuracy or latency) for lists with an increasing number of complements. If these effects can be observed, they will have to be explained in terms of decade effects on the level of individual additions. Specifically, we expect both post-complements [−] and complements [=] to be faster than additions crossing the next decade boundary [>], as well as additions not reaching the next decade [<]. This last prediction is of particular interest, as it would work against the influence of problem size (as complements on average involve larger addends and sums than sub­complements). Overall, we hypothesize that *addition type* explains the accuracy and speed of serial additions beyond the *problem-size* effect.

### (c) Results

We first analyse participants’ performance on the level of adding lists of single-digit addends, before focusing on the more fine-grained level of individual addition latencies. To render all effects transparent, we report error rates and latencies in absolute metrics, but log-transforming skewed variables yields qualitatively identical results.

#### (i) Adding lists

*Accuracy* As our serial addition paradigm allowed for a variety of errors, a conservative measure of accuracy distinguishes between lists which were correctly added on their initial presentation and those which induced at least one error. Table 2b shows the mean likelihoods of entering an erroneous result on a single list presentation by *list type*.

**Table 2.** Mean problem size measures, proportion of erroneously added lists and list addition latency (in s) by *list type*. Cell values denote the means and standard errors (in parentheses) of 210 observations.

*list type*	(a) number of elements	list sum	(b) error rate^a^	(c) latency^b^
*no* complements	4.92 (0.06)	24.5 (0.44)	23.3% (2.93%)	5.61 (0.16)
*one* complement	5.06 (0.05)	25.1 (0.41)	17.1% (2.61%)	4.93 (0.15)
*two* complements	5.23 (0.05)	25.7 (0.38)	4.8% (1.47%)	4.29 (0.12)

^a^ Proportion of genuine errors (excluding late entries) on a single list presentation.

^b^ Time needed to correctly add the entire list (in s) after excluding 11 outliers (1.75% of 630 lists, exceeding the overall mean by more than 3 s.d.).

Planned comparisons between the marginal means of a binomial regression model indicate that participants were not significantly more accurate on lists containing one complement than on lists containing no complements ($b_{1\;versus\;0}=0.41,OR=1.50,p=0.233$), but were systematically more accurate on lists containing two complements ($b_{2\;versus\;0}=1.87,OR=6.50,p<0.001$ and $b_{2\;versus\;1}=1.46,OR=4.32,p<0.001$, using Tukey’s adjustment for pairwise comparisons). Thus, lists with two complements elicited fewer errors.

*Latency* Only the latencies of correctly added lists were analysed. In an initial data screening procedure, we identified and excluded 11 outlier trials (exceeding the mean latency by more than 3 s.d.). Table 2c shows the mean addition latencies of the remaining 619 lists as a function of *list type*. Planned comparisons show that lists containing either one or two complements were added significantly faster than lists with no complements ($b_{1\;versus\;0}=0.77\;s,\eta_{p}^{2}=0.03,p<0.001$ and $b_{2\;versus\;0}=1.41\;s,\eta_{p}^{2}=0.10,p<0.001$, respectively) and lists with two complements were added significantly faster than lists with one complement ($b_{2\;versus\;1}=0.64\;s,\eta_{p}^{2}=0.02,p<0.001$, using Tukey’s adjustment for pairwise comparisons). As our list construction anticipated alternative explanations by controlling for the number and sum of addends (cf. table 2a), we can conclude that the presence of complements decreases the time needed to add lists of numbers.

#### (ii) Individual additions

According to our task analysis, the facilitative effects just reported could be attributed to *two* distinct and non-exclusive potential benefits of lists with complements: not only might the occurrence of a complement [=] itself facilitate calculation, but the effects may also be due to the post-complement [–] occurring immediately after a complement. To explain the observed benefits on a micro-level, we now compare the latencies of individual additions. As their elements are only known when participants report the correct sum, we only consider latencies of correct trials. As the frequency of addition types varied between lists and participants, we will note the total number of cases N on which summary values are based. After excluding 67 outliers (2.1% of 3195 additions, exceeding the overall mean by more than 3 s.d.), our analysis includes 3128 individual additions.

**Figure 1.**
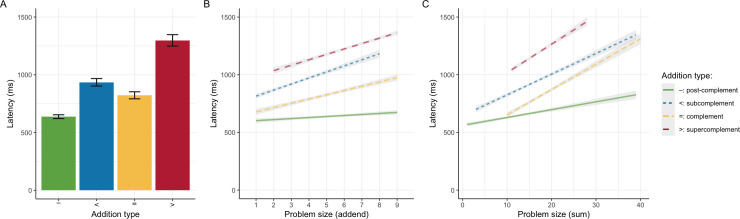
Mean addition latencies by *addition type* (A; error bars provide 95% confidence intervals) versus their predicted model trends by *problem size*, operationalized as *addend* (B) or *sum* (C).

Figure 1A shows the mean addition latencies by *addition type*. As the error bars denote 95% confidence intervals, it is obvious that post-complements [–] were significantly faster than all other types and complements [=] were significantly faster than sub­complements [<] and super­complements [>] (p<0.001 for all planned comparisons). Although this pattern corresponds to our hypothesized facilitation effects, an important question about these findings is whether they are merely problem-size effects in disguise. As higher addends are more likely to reach or cross the next decade boundary, our classification of addition types is to some extent confounded with problem size.

The interplay of *addition type* and problem size is non-trivial. To explore this issue, table 3a provides three plausible indicators of average problem size for each *addition type*. This clarifies that neither facilitation effect can be attributed to addend size, as sub­complements [<] have lower average addend sizes. Whereas the speed advantage of complements [=] over sub­complements [<] cannot be due to mean augend or sum sizes either, the advantage of post-complements [–] could be attributed to smaller augends or sums. Similarly, the slower addition times of super­complements [>] could be attributed to higher average addend or sum sizes.

**Table 3.** Mean *problem size* indicators and latency (in ms) of individual additions by *addition type*. Cell values denote the means and standard errors (in parentheses) of *N* observations.

*addition type*		(a) augend Au	addend a	sum Au + a	(b) latency	N ^a^
post-complement	[–]	6.23 (0.26)	4.99 (0.08)	11.20 (0.26)	638(8.33)	1078
subcomplement	[<]	12.40 (0.28)	3.29 (0.07)	15.70 (0.29)	935 (16.46)	758
complement	[=]	12.65 (0.29)	4.90 (0.10)	17.60 (0.29)	823 (15.56)	630
supercomplement	[>]	12.27 (0.26)	6.59 (0.08)	18.90 (0.26)	1298 (25.23)	662

^a^ Excluding 67 outliers (2.1% of 3195 additions, exceeding the overall mean by more than 3 s.d.).

To statistically disentangle the impact of *addition type* from problem size, we can operationalize the latter as *addend size*. When comparing a 2-factorial model that contains both *addition type* and *addend size* to baseline models that only contain either factor, the 2-factor model provides a significantly better fit to the data [$\chi^{2}(1)=104$ and $\chi^{2}(3)=921$, both p<0.001], but including *addition type* explains more incremental variance than including *addend size* ($\text{pseudo-}R^{2}=0.26\;\text{versus}\;0.03$, respectively). An exploratory model that incorporates the interaction between *addition type* and *addend size* provides an even better fit [$\chi^{2}(3)=63,p<0.001$], but explains little incremental variance ($\text{pseudo-}R^{2}=0.02$; figure 1B). Thus, the qualitative effects of *addition type* are modulated by *addend size*. An alternative operationalization of problem size as *sum* yields an analogous pattern of results (figure 1C).

Overall, the speed differences *between* addition types are as big as the differences *within* addition types as problem size increases. Thus, although *addition type* effects are clearly moderated by problem size, they cannot be reduced to them. (Figures 6–8 in appendix Ac provide alternative perspectives on this result.)

### (d) Discussion

Lists containing complements are added faster and with fewer errors. This may be due to a double benefit: both complements and subsequent post-complements are added faster than sub- and supercomplements, which have no round numbers as their augends or sums.

The fact that complements are added faster than sub­complements seems to go against the problem-size effect. However, our findings in no way contradict the more general phenomenon, which indeed our study has replicated in the serial addition paradigm. We conclude that *addition type* and *problem size* jointly determine addition latency.

The main finding of this first study illuminates the difficulty structure of tasks in serial additions. But demonstrating decade effects in mental addition does not yet explain *why* they occur. Study 2 will address this issue in a similar task that introduces a modicum of strategic choice.

## 4. Study 2

Having shown the existence of decade effects in a task involving the serial addition of single digits, we now move on to investigate the consequences of these effects in tasks where participants have some discretion about the order in which numbers are added. Study 2 explores a minimal change to the previous task by presenting *two* addends at a time. This allows participants a modicum of strategic choice by choosing how these addends are added to the running total. Our interest here lies in both replicating decade effects in this slightly enriched task environment and in seeking first evidence that participants will adaptively exploit the discretion they have over combining addends so as to benefit from the task difficulty gradients reported in study 1.

### (a) Task analysis

An extended serial addition paradigm involves adding the sequence $Au+a_{1}+a_{2}$, where Au is an internally represented augend and $a_{1}$ and $a_{2}$ are simultaneously presented external single-digit addends between 1 and 9. Adding two addends to an augend yields a surprisingly complex scenario. Responsible for this are two arithmetic laws which enable three distinct ways of combining two addends with an augend:


$$\begin{array}{rlr} Au+a_{1}+a_{2} & =(Au+a_{1})+a_{2} & \left(linear\;sequence\right)\\ & =Au+(a_{1}+a_{2}) & \left(by\;associativity\right)\\ & =(Au+a_{2})+a_{1} & \left(by\;commutativity\right). \end{array}$$

Extending our analysis of the single addend case, table 4 provides an overview of nine qualitatively distinct addition types for tasks in this form. This classification and the corresponding notation identify the *simplest possible* sequence to serially add the numbers $Au+a_{1}+a_{2}$, based on noting features of the task and applying arithmetic laws to seek and benefit from simpler addition problems (see appendix Ba for a more comprehensive analysis).

**Table 4.** Classification of *addition types* for adding two single-digit addends $a_{1}$ and $a_{2}$, $a_{1}\neq a_{2}$, to an augend Au (u denoting the augend’s unit). This typology and notation extends the *addition types* for adding single addends, as defined in table 1. (See table 7 in appendix Ba for a more comprehensive analysis.)

*addition type*			task features					example
category	name		notation	$u+a_{1}$	$u+a_{2}$	$a_{1}+a_{2}$	$u+a_{1}+a_{2}$	
(a) round augend ( u=0 )								
[-]	1. *decade-subcomplement*		[-<]	<10	<10	<10	<10	10+4+5
	2. *decade-complement*		[-=]	<10	<10	=10	=10	10+4+6
	3. *decade-supercomplement*		[->]	<10	<10	>10	>10	10+4+7
(b) non-round augend ( u>0 )								
[<]	4. *subcomplement*		[<<]	<10	<10	<10	<10	15+1+2
	5. *covert complement*		[<=]	<10	<10	<10	=10	15+2+3
[=]	6. *overt complement and pair:*							
	a*. direct complement*		[=-]	=10	≠10	≠10	>10	15+5+4
	b*. indirect complement*		[=-]	≠10	=10	≠10	>10	15+4+5
	c*. pair*		[=-]	≠10	≠10	=10	>10	12+6+4
	d*. direct complement-pair*		[=-]	=10	≠10	=10	>10	14+6+4
	e*. indirect complement-pair*		[=-]	≠10	=10	=10	>10	14+4+6
[>]	7. *supercomplement*		[<>]	≠10	≠10	≠10	>10	15+2+4
	8. *covert complement 2*		[>=]	>10	>10	>10	=20	18+5+7
	9. *supercomplement 2*		[>>]	>10	>10	>10	>20	18+5+8

The first three addition types all begin with a round augend (i.e. u=0) and either fail to add up to [− <], precisely reach [− =] or cross [− >] the next decade boundary. By contrast, addition types 4−9 do not start with a round augend (u≠0) and span up to two decades. Types 4, 7 and 9 have in common that they do not involve any round numbers but differ in crossing no [< <], one [< >] or two [> >] decade boundaries, respectively. By contrast, types 5, 6 and 8 all allow for round numbers *if* chosen in a specific order. For additions of types 5 and 8, the sum of both addends complements the current augend to the next [− =] or second-next [− =] decade, but neither addend directly complements the current augend. As the complement only becomes apparent once one addition has been made, we refer to these cases as *covert* complements. In contrast, *overt* complements and *pairs* (type 6) are defined by either one of the addends complementing the current augend (6a and 6b), by both addends complementing each other to 10 (i.e. forming a *pair*, 6c) or by both (6d and 6e).

On a finer level, the cases of types 6a–6e have in common that two out of the three relevant elements (u, $a_{1}$ and $a_{2}$) add up to 10, which means that they can be added as a sequence of a complement and a post-complement [= −]. They differ with respect to the particular order in which they need to be added to take advantage of this feature. Of particular interest is the distinction between *direct* and *indirect* types of complements and complement-pairs. If indirect types (6b and 6e) were added as quickly as their direct counterparts (6a and 6d), this would suggest a reversal of addend order.

Importantly, this typology assumes the validity of our analysis in study 1 and the adaptive application of arithmetic laws to benefit from difficulty gradients in mental operations. Whether people spontaneously detect easier addition types and thus take advantage of ‘mental shortcuts’ to exploit facilitative opportunities is the empirical question addressed by this study.

### (b) Method

The key change in study 2 involved the simultaneous presentation of two addends (as opposed to one in study 1). Due to this change, the instructions now explicitly mentioned that the numbers can be added ‘in any order,’ and again laid an emphasis on both accuracy and speed. As our general methodology was described in §2, we only provide study-specific details here.

Study 2 employed a fully within-subjects design, with *list type* (allowing for *no*, *some*, versus *many* pairs and complements) being the main experimentally controlled factor. The second factor, *addition type* (table 4) was randomized within participants.

As in study 1, random lists of single-digit numbers were generated on the fly. Each participant faced a unique set of 30 lists of 8, 10 or 12 single-digit numbers (1–9) with no repetitions of adjacent elements. Each list belonged to one of three sets (*list type* s), which were comparable in their number of elements and total sums, but differed with respect to their number of potential facilitative opportunities: 10 lists allowed for *no* complements or pairs (e.g. 45, 34, 96, 43); 10 lists allowed for *some* complements and pairs (one of each, e.g. 61, 43, 82, 94); and 10 lists allowed for *many* complements and pairs (three or more, e.g. 84, 28, 53, 91). To be comparable yet pre­empt alternative explanations, the number of list elements and sums per set satisfied additional constraints (see appendix Bb for details).

On the level of lists, we expect that lists enabling more complements and pairs are added with higher accuracy and speed than those enabling fewer facilitators. On the level of individual additions, we aim to replicate key findings from study 1 for a more complex task. When grouping our fine-grained analysis of addition types into four categories corresponding to the types of study 1 (see the first column of table 4), we expect the same pattern of latencies (i.e. additions with round augends and involving complements and pairs are added faster than others). And while *problem size* is likely to be a key factor for addition latency, we propose that our *addition type* category also makes a systematic contribution and may interact with problem size to explain the latency data.

Beyond these replications, we hope to gain insights into the mechanisms of mental addition by inspecting the pattern of latency results for the nine postulated addition types. If participants primarily benefit from complements, all addition types involving them should be faster than those that do not. By contrast, a systematic advantage of the addition types that enable the co-occurrence of a complement and its subsequent post-complement would assign an auxiliary role to complements.

As we cannot know the search costs of detecting addition types and reversing addend orders, we will explore latencies with regard to effects of order and opportunity. If the linear order of addends is mostly preserved, we would see higher latencies for indirect complements and pairs than for direct ones. Alternatively, similar latencies for direct and indirect complements and pairs would suggest strategic choices for processing addends in preferred orders and support an adaptive account. Finally, we expect to observe effects of opportunity. Adders may generally benefit from difficulty gradients but also incur some cost for finding optimal sequences. If adders seize any opportunity for reaching a round number, addition types with multiple ways to yield a complement or pair (e.g. types 6d and 6e) will be faster than those offering fewer opportunities.

### (c) Results

As in study 1, we first analyse participants’ accuracy and latency on the level of lists, before considering the latencies of individual additions.

#### (i) Adding lists

*Accuracy* The accuracy of adding lists is conservatively measured by the proportion of correct entries on each list’s first presentation. Table 5b shows the mean error rates by *list type*, which were generally higher than those for adding just single addends (cf. study 1, table 2b). As hypothesized, the mean frequency of errors was highest (29.0%) for lists allowing for no pairs or complements and decreased (to 21.9% and 20.0%) as the number of possible pairs and complements within the stimulus lists increased. But despite these trends in the predicted direction, the planned comparisons between the marginal means of a binomial regression model were non-significant ($b_{some\;versus\;no}=0.39,OR=1.48,p=0.201$, $b_{many\;versus\;no}=0.51,OR=1.67,p=0.073$ and $b_{many\;versus\;some}=0.12,OR=1.13,p=0.876$, using Tukey’s adjustment for pairwise comparisons). Thus, lists enabling complements or pairs yielded no systematic benefits in addition accuracy.

**Table 5.** Mean problem size measures, proportion of erroneously added lists, and list addition latency (in s) by *list type*. Each cell contains the means and standard errors (in parentheses) of 210 observations.

*list type*	(a) number of elements	list sum	(b) error rate^a^	(c) latency^b^
*no* complements or pairs	9.79 (0.11)	49.5 (0.71)	29.0% (3.14%)	18.9 (0.50)
*some* complements and pairs	10.03 (0.11)	50.2 (0.69)	21.9% (2.86%)	15.3 (0.42)
*many* complements and pairs	10.26 (0.10)	51.3 (0.62)	20.0% (2.77%)	13.8 (0.37)

^a^ Proportion of genuine errors (excluding late entries) on a single list presentation.

^b^ Total time needed to *correctly *add the entire list (in s) after excluding five outliers (0.79% of 630 lists, exceeding the overall mean by more than 3 s.d.).

*Latency* Table 5c shows that mean latencies for correctly adding lists of a particular *list type* also matched the hypothesized trend. Planned comparisons confirm that participants added both lists allowing for *some* and *many* pairs and complements significantly faster than lists allowing for *no* pairs and complements ($b_{some\;versus\;no}=3.56\;s,\eta_{p}^{2}=0.08$ and $b_{many\;versus\;no}=5.09\;s,\eta_{p}^{2}=0.15$, respectively, both p<0.001) and those allowing for *many* pairs and complements still faster than those with *some* pairs and complements ($b_{many\;versus\;some}=1.53\;s,\eta_{p}^{2}=0.02,p=0.006$, using Tukey’s adjustment for pairwise comparisons). Thus, lists providing more opportunities for pairs and complements are added faster than those with fewer such facilitators.

#### (ii) Adding pairs of addends

To *explain* the benefits of lists containing pairs and complements, but also to assess the validity of our hypotheses concerning participants’ utilization of ‘mental shortcuts’ (by facilitating additions through adding numbers in an easier order), we need to analyse the adding latencies within lists, i.e. the duration of individual pairwise additions. On this level, only latencies of correct trials can be meaningfully analysed. A data screening procedure identified 45 outliers (1.42% of 3158 pairwise additions, exceeding the overall mean by more than 3 s.d.), which were excluded from all further analyses.

**Figure 2.**
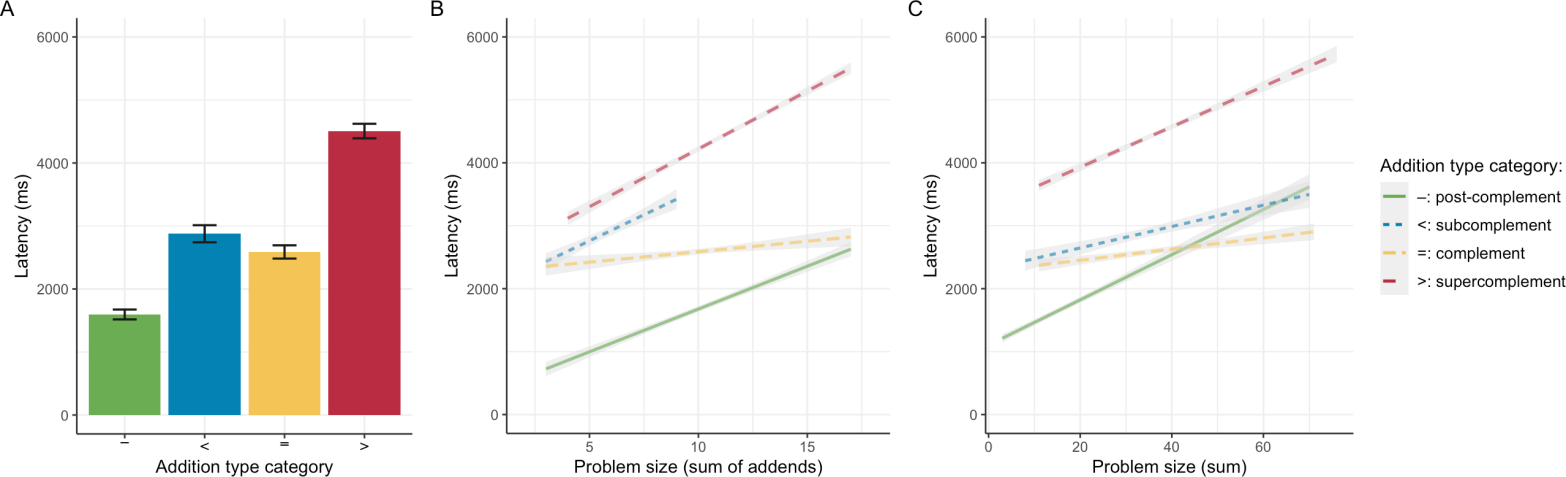
Mean addition latency by *addition type category* (A; error bars provide 95% confidence intervals) versus their predicted model trends by *problem size*, operationalized as addend sum (B) or problem sum (C).

When grouping *addition types* into the four original categories of study 1 (table 1), we can replicate its key results. Despite generally higher latencies, figure 2A shows the same pattern of latency differences as figure 1A. As before, it is clear that post-complements [−] were significantly faster than all other addition types and complements [=] were significantly faster than sub­complements [<] and super­complements [>] (using Tukey’s adjustment for pairwise comparisons, the speed advantage of complements over sub­complements is significant at p<0.01, all other comparisons at p<0.001).

Statistical models that examine the interplay of *addition type* category with measures of problem size yield analogous results to those obtained before (compare figure 2B,C to figure 1B,C). A 2-factorial model that includes the *addition type* category and the *addend sum* as an indicator of problem size provides a better fit than either factor by itself ($\chi^{2}(1)=298$ and $\chi^{2}(3)=1466$, respectively, both p<0.001) but including the *addition type* category explains more incremental variance than including *addend sum* ($\text{pseudo-}R^{2}=0.38\;\text{versus}\;0.09$, respectively). As in study 1, an exploratory model that includes the interaction between both factors provides an even better fit ($\chi^{2}(3)=46,p<0.001$), but explains little incremental variance ($\text{pseudo-}R^{2}=0.01$, see figure 2B for the predicted model trends). As before, the alternative operationalization of problem size as *sum* yields an analogous pattern of results (figure 2C). Thus, the effects of *addition type* category and indicators of problem size interact with each other and jointly explain addition latency.

**Figure 3.**
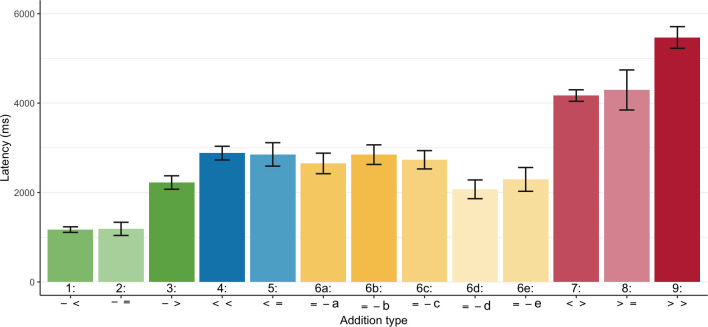
Mean addition latency by *addition type* for additions of two addends (as defined in table 4. Error bars provide 95% confidence intervals).

**Figure 6.**
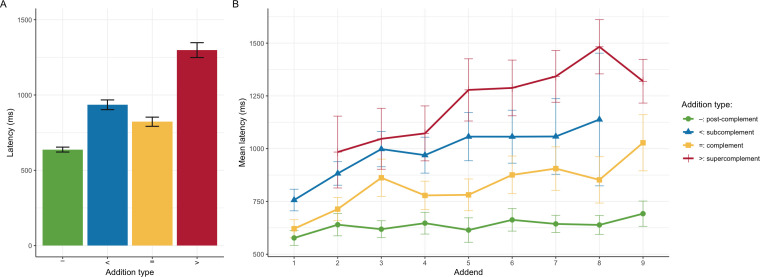
(A) Mean addition latencies by *addition type*. (B) Empirical trends for the interaction of *addition type* and *problem size* (operationalized as addend size). (Error bars in both panels provide 95% confidence intervals. Note that the y-axis of panel B is truncated.)

**Figure 7.**
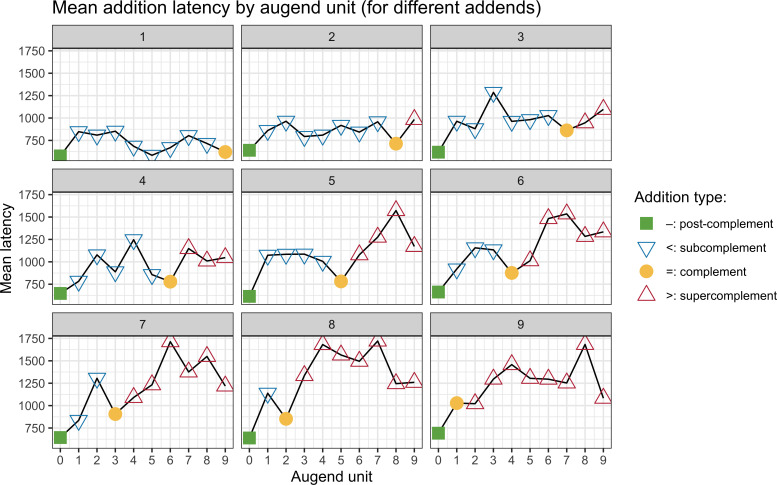
Mean addition latencies of correct additions by augend unit for single-digit addends. Each panel depicts the mean latencies for a specific addend a by augend units u. The panels 1–9 illustrate the following effects: Minimal latencies for most addends at u=0 indicate the post-complement advantage. Local latency minima at $u_{c}=10-a$ (as indicated by the yellow symbol in each panel) signal the complement advantage. Shorter latencies for augend units $u<u_{c}$ than for $u>u_{c}$ are due to subcomplements being faster than supercomplements. The generally increasing latency of values both between panels (with increasing addends) and within panels (with increasing augend units) signal problem size effects, which appear to be exacerbated both by larger addends and larger augend units.

**Figure 8.**
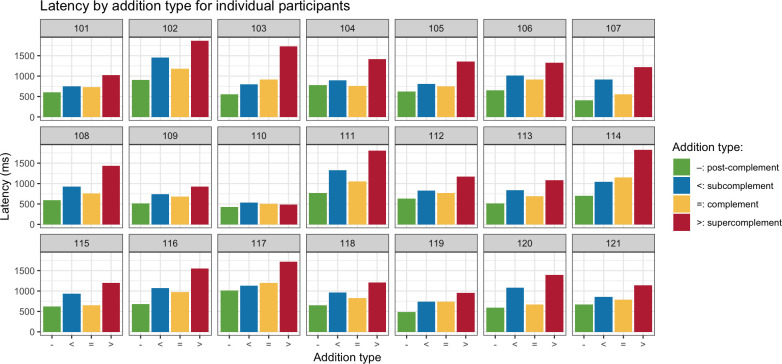
Mean addition latencies by *addition type* for each of the 21 participants. Although some individuals show much stronger effects than others (e.g. compare ID=102, 111 or 120, with ID=110, 119 or 121), the general pattern is remarkably consistent across participants.

To assess our specific hypotheses concerning strategic addend choices, figure 3 shows the mean addition latency for our more fine-grained *addition type* analysis. Corresponding to the aggregated view of figure 2A, additions with round augends (types 1−3) are faster than those with non-round augends (types 4−9), and types 7−9, which cross over one or two decade boundaries, are relatively slow. As expected, decade-super­complements [−>] (type 3) and super­complements 2 [> >] (type 9) are systematically slower than the other addition types within their category, but this may partly be due to their higher addend sums (see table 8a, appendix Bc for problem size indicators for each *addition type*). It does not require any statistical test to see that decade-complements [− =], covert complements [< =] and covert complements 2 [> =] (i.e. types 2, 5 and 8) were *not* faster than their corresponding sub­complements (types 1, 4 and 7). In contrast, additions allowing for both a complement and a post-complement exhibit clear facilitation effects. Within overt complements and pairs [= −] (type 6), indirect complements (type 6b) and indirect complement-pairs (type 6e) are only marginally slower than their direct counterparts (types 6a and 6d). However, their substantial speed-up relative to super­complements [< >] (type 7) demonstrates that they often must have been chosen in the preferred order of their types (for otherwise additions of types 6b and 6e would be equal to those of type 7). Finally, the boosts in speed for adding complement-pairs (types 6d and 6e) show that addition types with multiple opportunities for facilitations benefit from effects of opportunity. (To safeguard against alternative explanations, table 8a in appendix Bc provides several indicators of problem size.)

### (d) Discussion

The minor extension of our initial paradigm to pairs of addends yielded a rich pattern of results. The finding that overt complements and pairs are added faster than sub­complements replicates the most important result of study 1 and, at the same time, suggests that participants are sufficiently sensitive to minor difficulty gradients in mental additions that they adjust their strategies to take advantage of these differences.

A surprising finding is the lack of a general speed-up of complements over sub­complements. As complements occurring in combination with an immediate post-complement *are* added faster, however, the key benefit may arise from the latter. This suggests that the motivation for seeking complements in an addition process may be their auxiliary role in enabling subsequent post-complements.

The fact that indirect addition types are processed about as fast as direct ones suggests strategic reversals of addend order. Although small speed-ups would alternatively be explained by a model that assumes a random choice of addends, it is implausible that such a non-adaptive account would yield effects of the observed magnitude. Despite this evidence for strategic and systematic choices, clear effects of opportunity point towards some variation in seeking and exploiting easier addition types.

## 5. General discussion

We have shown that our familiarity with Western numerals results in systematic variation across a range of simple addition types, defined in terms of decade boundaries. As suggested by prior research on carry effects [46,47], sums that cross decade boundaries (super­complements) are slower and more error-prone than sums that remain within decade boundaries (sub­complements). More surprisingly, sums that exactly reach decade boundaries (complements) are also quicker than sub­complements, even though this comparison works against the problem-size effect. Finally, we replicated that post-complements in which the augend is a decade boundary and pairs of addends adding up to 10 are the quickest additions of all [45].

Explaining the benefits of post-complements and pairs seems rather trivial. When using Western numerals, expressing their sums only requires notational interventions and can thus refrain from any proper addition (e.g. by counting or recalling facts). Adding a single-digit addend *a* to a round number is reduced to replacing 0 by *a*. Similarly, adding a pair of addends *a*1 + *a*2 = 10 to a number in this notation merely requires incrementing its decade unit by 1. But as participants were adding numbers in their heads, rather than on paper, this correspondence between internal and external operations is remarkable.

The benefits of complements over sub­complements are more elusive and more puzzling, especially as their occurrence usually defies the problem-size effect. While base-10 may be a cultural attractor [52], we posit that its adoption is the cause, rather than a consequence of the effects uncovered here. A possible explanation for their speedy and robust use is that complements are practiced more frequently than other additions. If adders routinely de­compose post-complements into a complement and its carry [15,30], complements occur with much higher frequency than subcomplements. One strand of support for this explanation is offered by the results of study 2. Here, we showed that when adding pairs of numbers, people will adjust the order of sums so as to take advantage of the faster processing times of complements and post-complements. Such a strategy in and of itself determines that complements will be practiced more, if one assumes that adders prefer round intermediate sums when adding numbers in everyday contexts. As such a bootstrapping account would fail to explain the initial advantage of complements, our findings suggest a more subtle role for them: rather than being ends in themselves, complements enable subsequent post-complements.

Because our studies lacked an independent measure of arithmetic ability, their implications for inter­individual differences remain speculative. Similarly, our focus on skilled adults limits our potential for explaining the development of arithmetic strategies. However, inspecting our data suggests the hypothesis that less proficient adders may exhibit stronger decade effects. If this is a stable pattern, a cognitive scaffolding account could argue for a trade-off between arithmetic skills and the interactive use of simplification strategies [53]. As our analysis neither depends on the particular base-10 nor on numbers being added, it could be generalized to number systems using non-decimal bases and other arithmetic operations. Empirical studies on this would also clarify the roles of familiarity and practice for the emergence of base effects.

With respect to theories of the internal representation of numbers, we hesitate to draw far-reaching conclusions. On the surface, our findings appear to favour a decomposition model for the representation of multi­symbol numbers [21]. More specifically, decade effects in mental addition could motivate an account that argues for ‘decade breaks’ in the sense of encoding the digits of units and decades of multi­symbol numbers in separate bins, rather than on a continuous number line [54,55]. Although we are sympathetic to such accounts, our serial addition paradigm deviates from the number comparison tasks used in these contexts and may be ill-suited to settle corresponding debates. An interesting aspect of our paradigm is that very few additions require any output of their sum. Rather, these sums are held in mind as the augend for the next addition. This makes our findings of a complement advantage even more striking, because there is no reason for mentally representing round augends as Western numerals, except that our inputs and outputs are using this format. Indeed, we suspect that intermediate values are represented linguistically, in that people are likely to rehearse as they add. This means that we have documented systematic decade effects in an addition task not requiring this particular base and the use of Western numerals. As our typology only hinges on the properties of place-value notations, further studies must clarify whether these effects generalize to other base values (e.g. 8 or 16), arithmetic tasks (e.g. serial subtraction), sensory modalities (e.g. auditory stimuli) and numeric representations (e.g. using non-Western symbols).

Our caution against drawing premature conclusions regarding internal representations partly stems from Marr’s distinction of three levels of analysis [56]. We believe our findings are best explained on the computational and algorithmic, rather than on the implementational level. While our effects show that mental calculations are bounded and constrained by underlying representations, they also illustrate their flexibility and ingenuity. This ambiguity highlights the cyclical nature of our analysis. Whatever the detailed explanation for the gradients of difficulty we have exposed, they exemplify an important relation between external representations and internal processes. Decade effects demonstrate how conventional, but essentially arbitrary properties of an external representation (i.e. the base-10 place-value notation of Western numerals) impact primitive internal operations. This, in turn, impacts the strategies by which primitive operations are chosen and combined, with implications for effects of relative difficulty and practice. When studying this cyclical interplay in interactive task environments, adaptive strategies give rise to new ways of coping with and over­coming representational constraints (e.g. by pointing at or moving objects; see [57,58]). Adopting an embodied and embedded perspective on these dynamics not only reveals new aspects of numerical cognition [59,60], but highlights the intimate relation between the mind and its technologies.

## 6. Conclusion

Our studies of serial addition have documented a rich landscape of decade effects. Their emergence in consistent patterns demonstrates that one foundation for the mechanics of mental addition is quite material: while the fabrics of our mental devices are not constrained by metal gears, our number sense is anchored in the notational technology of the Western numerals.

Fortunately, the constraints imposed by any representation need not be passively endured. Instead, the subtle difficulty gradients imposed by choosing a particular base in a place-value system can actively be exploited by creating simpler sequences of operations. Even in minimally interactive contexts, solving mental arithmetic problems involves strategic aspects that distribute the computational process over the mind and its environment (see the theoretical notions of *situated actions*, *distributed cognition* and the *functional task environment*, in [61–63]).

Overall, the mental gears of our arithmetic minds face the same constraints as Babbage’s analytical engine. Just as the design of his machine required some special ingenuity for carrying the tens, experienced adders have developed intricate ways for tailoring their arithmetic tasks to decade boundaries. Ironically, contemporary efforts for finally building Babbage’s engine must first decipher his mechanical notation [64]. Thus, any cultural quest for suitable number systems is not only constrained by the mathematical properties of numbers, but must design systems that also meet our computational and practical demands. Different notations can render the same problem easy or difficult, and our cognitive adaptations to notational constraints fundamentally shape how we think about and deal with numbers.

## Figures and Tables

**Figure 4.**
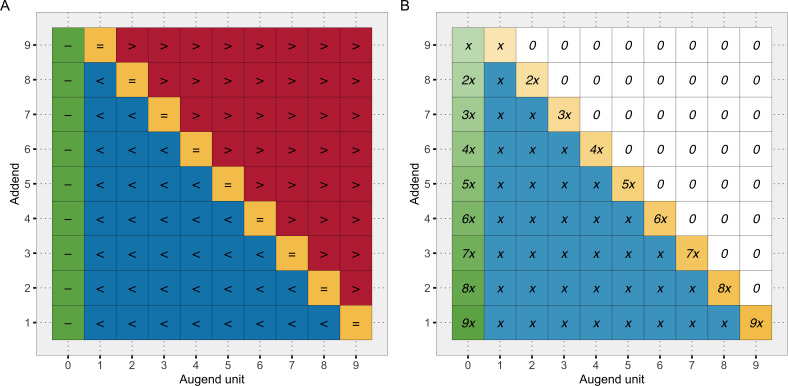
(A) The distribution of *addition types* when adding single-digit addends a (from 1 to 9) to an augend unit u (from 0 to 9). (B) Predicted proportion of addition types if any supercomplement [>] is decomposed into a complement [=] and a post-complement [−]. For x=1/(90+36)≈.79%, all proportions sum to 100%.

**Figure 5.**
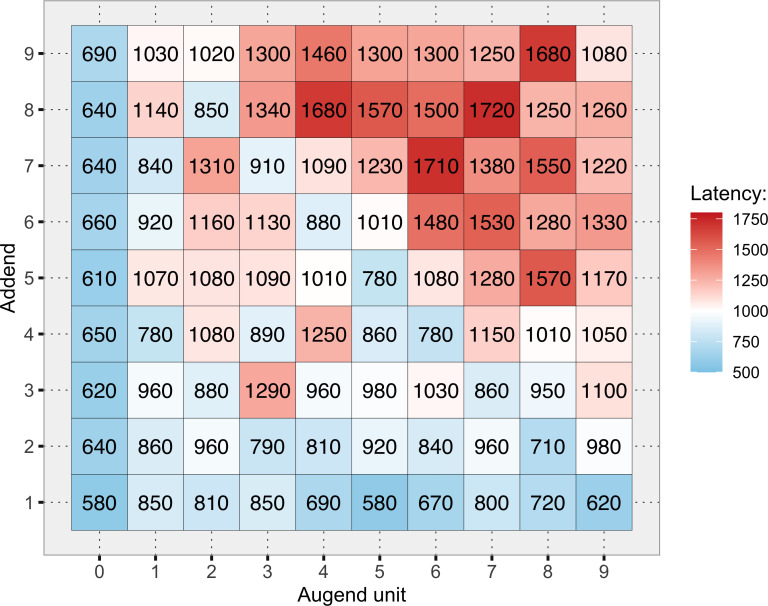
Mean observed latencies for adding single-digit addends a (from 1 to 9) to an augend unit u (from 1 to 9). The colour gradient indicates differences in addition latencies.

**Figure 9.**
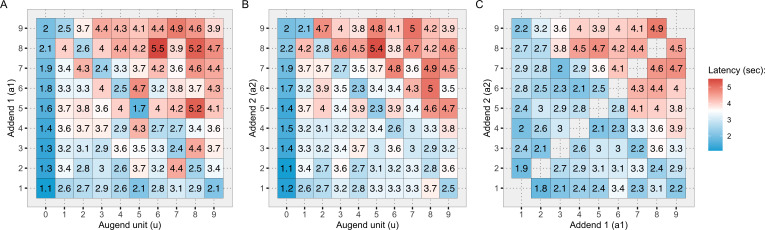
Mean observed addition latency by *augend unit* and *addend size* ($a_{1}$ or $a_{2}$). Each panel collapses across one dimension of a 9×9×10 cube. The colour gradient indicates differences in addition latencies

## Data Availability

The raw data and all analysis scripts are available at: [65].

## Appendix A. Study 1

### A.1. Task analysis

Assume we randomly draw single-digit addends a from a uniform distribution a∈{1…9} and classify the additions that yield their cumulative sums into the four addition types of table 1. Any super­complement [>] can then be de­composed into a complement [=] and a post-complement [−]. Table 6 provides the expected proportion of addition types *without* and *with* decomposition of all super­complements. Whereas *complements* and *post-complements* are relatively rare (10% each, without decomposition), their relative frequency increases when *super­complements* are decomposed (up to 35.7% each, with complete decomposition).

**Table 6.** The proportion of *addition types* for adding a random single-digit addend a (drawn from a uniform distribution a∈{1…9}) to an augend Au (u denoting the augend's unit) with and without decomposing supercomplements.

*addition type*				proportion	
name	notation	u	u+a	*without* /	*with* decomposition:
*post-complement*	[−]	=0	<10	10.0	35.7
*sub-complement*	[<]	>0	<10	40.0	28.6
*complement*	[=]	>0	=10	10.0	35.7
*supercomplement*	[>]	>0	>10	40.0	—

Figure 4 provides a spatial perspective on this argument. Assuming a uniform distribution of single-digit addends a (from 1 to 9), the resulting *addition types* are distributed systematically over the space of addends a by augend units u (from 0 to 9). Figure 4A shows the distribution of addition types for all possible combinations a×u.

If any super­complement [>] is de­composed into a complement [=] and a post-complement [−], we obtain (by combinatorics or by simulation) the distribution of additions shown in figure 4B. Note that the frequency of complements [=] increases with higher augend unit u and higher addends a, whereas the frequency of post-complements [−] is inversely proportional to the magnitude of addends a. Any variation in addition strategies (e.g. partial decomposition) would diminish the systematicity of this pattern. Similarly, actually computing the decomposition parts may involve additional computations. (See figure 5 below for an illustration of the mean observed addition latencies.)

### A.2. Material and Methods

To maximize the variability of lists and additions, each participant received a unique set of 30 lists, which were computer-generated during the experiment. Each list consisted of four to six single-digit numbers from 1 to 9, without any repetitions of adjacent elements. Through this method of generation, materials were controlled at the level of *list type*, but the particular elements within lists (addends, augends) varied randomly within pre-specified constraints. Three *list types* of 10 lists each were distinguished:

(1) Lists in set 1 contained *no* complements (e.g. 2,9,4,2,6,4);
(2) lists in set 2 contained exactly *one* complement (e.g. 2,1,4,3,8); and
(3) lists in set 3 contained exactly *two* complements (e.g. 6,4,2,7,1).

To render the three sets of lists comparable, both their total number of elements n and the total sum s of their elements were constrained to deviate less than 10% from their expected random values of 50 and 250 (46≤n≤54, 230≤s≤270), respectively.

To pre­empt any alternative explanations of the hypothesized effects, all remaining differences went contrary to a potentially facilitative influence of complements. Thus, for every participant, any set i of lists with (one or two) complements contained at least as many elements $n_{i}$ and as high a total sum $s_{i}$ as any set of lists with fewer complements, i.e. $n_{1}\leq n_{2}\leq n_{3}$ and $s_{1}\leq s_{2}\leq s_{3}$.

### A.3. Results

Figures 5–8 provide additional and alternative perspectives on the reported results:

— Figure 5 shows the overall mean observed latencies for adding single-digit addends a (from 1 to 9) to an augend unit u (from 1 to 9). Despite some fluctuations, the colour gradient indicating differences in addition latencies corresponds closely to the qualitative pattern of *addition types* (as shown in figure 4).
— Figure 6 contrasts mean addition latencies by *addition type* (A) with their trends when viewed as a function of *problem size* (operationalized as *addend size* B). (Note that figure 6A corresponds to figure 1A.)
  Whereas the speed benefit of complements [=] over sub­complements [<] appears to contradict the problem-size effect, *addition type* and problem size interact with each other.
  To deconfound both effects graphically, figure 6B displays the mean observed latencies for adding addends 1 to 9 separately for each addition type. This visualization shows hardly any addend size effect for post-complements [−], a small addend size effect for complements [=], as depicted by the steeper slope of the line with increasing addend size, and stronger addend size effects for sub- [<] and super­complements [>].
  However, the difference *between* addition types for many addends is as big as the differences *within* addition types as addend size increases. Thus, while *addition type* effects are moderated by addend size, they cannot be reduced to them.
— Figure 7 shows mean addition latency as a function of the augend unit u, separately for each addend a (ranging from 1 to 9, in different panels). This further illustrates the combined effects of addend size a and augend unit u (as two determinants of problem size) on addition latency.
— Figure 8 shows the mean latencies of each *addition type* separately for each individual participant (in different panels). This illustrates that the qualitative pattern observed for aggregate data also holds for a majority of individuals (though note some exceptions, e.g. ID=110).

## Appendix B. Study 2

The extended serial addition paradigm used in study 2 involves repeatedly adding problems of the form $Au+a_{1}+a_{2}$, where the current augend Au is mentally represented and two addends $a_{1}$ and $a_{2}$ are displayed simultaneously on the screen. For $a_{1}\neq a_{2}$, the range of possible addend sums is $3\leq (a_{1}+a_{2})\leq 17$.

### B.1. Task analysis

The following task analysis identifies the simplest possible addition types for the task of adding two single-digit addends $a_{1}$ and $a_{2}$ to a double-digit augend Au (A being the augend decade, and u being the augend unit). It thus justifies the terminology and notation introduced in the context of study 2.

Table 7 defines nine qualitatively different *addition types*. The first three types all start at a decade boundary, i.e. with a round augend (u=0):

(1) *decade-subcomplement*: if the sum is lower than 10 ($a_{1}+a_{2}<10$), the result for additions of type [−<] stays within the current decade.
(2) *decade-complement*: if both addends complement each other, they form a *pair* ($a_{1}+a_{2}=10$). Thus, additions of problem type [− =] begin and end on a round number.
(3) *decade-supercomplement*: if the sum of both addends exceeds 10 ($a_{1}+a_{2}>10$), the result of additions of type [−>] necessarily cross the next decade boundary.

**Table 7.** Classification of *addition types* for the addition of two single-digit addends $a_{1}$ and $a_{2}$ to a double-digit augend Au (u denoting the augend’s unit) in the base-10 place-value system of numeral notation. (See table 4 in §4(a) for a simplified version.).

*addition type*	task features				sequence of *addition types^a^*			example
	$u+a_{1}$	$u+a_{2}$	$a_{1}+a_{2}$	$u+a_{1}+a_{2}$	$(u+a_{1})+a_{2}$	$(u+a_{2})+a_{1}$	$u+\left(a_{1}+a_{2}\right)$	
(a) round augend ( u=0 )								
1 [−<]	<10	<10	<10	<10	[-<]	[-<]	[<-]	10+4+5
2 [−=]	<10	<10	=10	=10	[-=]	[-=]	[=-]	10+4+6
3 [−>]	<10	<10	>10	>10	[->]	[->]	[>-]	10+4+7
(b) non-round augend ( u>0 )^b^								
4 [<<]	<10	<10	<10	<10	[<<]	[<<]	[<<]	15+1+2
5 [<=]	<10	<10	<10	=10	[<=]	[<=]	[<=]	15+2+3
6a [=−]	=10	<10	<10	>10	[=-]	[<>]	[<>]	15+5+4
	=10	<10	>10		[=-]	[<>]	[><]	11+9+2
	=10	>10	>10		[=-]	[><]	[><]	15+5+6
6b [=−]	<10	=10	<10	>10	[<>]	[=-]	[<>]	15+4+5
	<10	=10	>10		[<>]	[=-]	[><]	11+2+9
	>10	=10	>10		[><]	[=-]	[><]	15+6+5
6c [=−]	<10	<10	=10	>10	[<>]	[<>]	[=-]	12+6+4
	<10	>10	=10		[<>]	[><]	[=-]	15+4+6
	>10	<10	=10		[><]	[<>]	[=-]	15+6+4
	>10	>10	=10		[><]	[><]	[=-]	18+6+4
6d [=−]	=10	<10	=10	>10	[=-]	[<>]	[=-]	14+6+4
	=10	>10	=10		[=-]	[><]	[=-]	16+4+6
6e [=−]	<10	=10	=10	>10	[<>]	[=-]	[=-]	14+4+6
	>10	=10	=10		[><]	[=-]	[=-]	16+6+4
6f [=−]	=10	=10	=10	>10	[=-]	[=-]	[=-]	15+5+5 ^c^
7 [<>]	<10	<10	<10	>10	[<>]	[<>]	[<>]	15+2+4
	<10	<10	>10		[<>]	[<>]	[><]	12+5+7
	<10	>10	<10		[<>]	[><]	[<>]	15+3+6
	<10	>10	>10		[<>]	[><]	[><]	14+5+8
	>10	<10	<10		[><]	[<>]	[<>]	15+6+3
	>10	<10	>10		[<>]	[<>]	[><]	12+7+5
	>10	>10	<10		[><]	[><]	[<>]	18+5+3
	>10	>10	>10		[><]	[><]	[><]	18+5+6
8 [>=]	>10	>10	>10	=20	[>=]	[>=]	[>=]	18+5+7
9 [>>]	>10	>10	>10	>20	[>>]	[>>]	[>>]	18+5+8

^a^ Addition types for additions of single addends are defined in table 1.

^b^ Most addition types impose additional constraints on the actual value of *u*. For example, for types 8 and 9 *u* needs to exceed 1, and for type 6d *u* needs to be equal to *a*2.

^c^ As the stimulus materials in study 2 contained no immediate repetitions (i.e. *a*1 ≠ *a*2) this case could not occur.

Types 4−9 do not start with a round augend (u≠0) and span up to two decades. Types 4, 7 and 9, which we call *sub­complements* [< <], *super­complements* [< >] and *super­complements 2* [> >], have in common that they do not involve any round numbers. They differ in crossing no, one or two decade boundaries, respectively. In contrast, types 5, 8 and 6, called *covert complements* [−=], *complements 2* [>=] and *overt complements and pairs* [=−], all allow for round numbers *if* chosen in a specific order. For additions of type [<=] and [>=], the sum of both addends complements the current augend to the next or second next decade. However, neither addend directly complements the current augend. As the complement only becomes apparent once one addition has been made, we refer to these cases as *covert* complements. In contrast, *overt* complements and pairs are defined by either one of the addends complementing the current augend or both addends complementing each other to 10 (i.e. forming a *pair*), or both.

On an even finer level, the possibly facilitative cases of type 6a−6e all have in common that two out of the three relevant elements (u, $a_{1}$ and $a_{2}$) add up to 10, which means that they can be added as a sequence of a complement and a post-complement [=−]. They differ with respect to the particular *order* in which they need to be added to take advantage of this feature. Specifically, the five subtypes of *overt complements and pairs* (type 6) can be distinguished further as follows:

(a) For *direct complements* the first addend $a_{1}$ complements the current augend unit u to 10. Numbers can be added *linearly*, i.e. in the order in which they are presented.
(b) In *indirect complements*, the second addend $a_{2}$ complements the current augend unit u to 10. To take advantage of this, participants need to add the right addend $a_{2}$ before the left addend $a_{1}$, thus implicitly applying the *commutativity* of addition.
(c) If both addends add up to 10, they form a *pair*. *Pairs* differ from complements in the fact that both numbers adding up to 10 are externally present. To take advantage of pairs means implicitly applying the *associativity* of addition.
(d) and e. Pairs and complements do not mutually exclude each other, as either addend of the pair can also complement the current augend unit.

A theoretically possible case (6f) of both addends forming a pair *and* complementing the augend—as in 15+5+5—was excluded by disallowing repetitions of adjacent addends.

### B.2. Material and Methods

As in study 1, random lists of single-digit numbers were generated on the fly. Each participant faced a unique set of 30 lists of 8, 10 or 12 single-digit numbers (1–9) with no repetitions of adjacent elements. Each list belonged to one of three sets (*list type* s), which were comparable in their number of elements and total sums, but differed with respect to their number of potential facilitative opportunities:

— *no* complements or pairs: 10 lists allowed for no complements or pairs (e.g. 45, 34, 96, 43);
— *some* complements and pairs: 10 lists allowed for exactly one complement and one pair each (e.g. 61, 43, 82, 94);
— *many* complements and pairs: 10 lists allowed for more than two complements or pairs each (e.g. 84, 28, 53, 91).

To render lists comparable and to prevent alternative explanations of the hypothesized facilitation effects, each set of lists satisfied the following constraints:

— the total number n of elements varied no more than 5% around the expected value of 100, i.e. 95≤n≤105;
— the sum s of all elements varied no more than 5% around the expected value of 500, i.e. 475≤s≤525;
— the total number n and sum s of elements in any set must not exceed those in sets with more facilitative opportunities.

### B.3. Results

The following materials provide further details on the results reported in §4c.

#### (i) Addition latency and problem size

Table 8 shows the mean latency for pairwise additions by fine-grained *addend type*. Whereas figure 3 illustrates only latencies, table 8a provides additional indicators of *problem size* and notes the number N of observations per type.

**Table 8.** Mean *problem size* indicators and addition latency (in ms, with standard errors in parenthesis) when adding two addends by *addition type*.

*addition type^a^*			(a) *problem size*			(b) latency	N ^b^
name		notation	Au	$a_{1}$ + $a_{2}$	Au + $a_{1}$ + $a_{2}$		
(a) round augend ( u=0 )							
1. *decade-subcomplement*		[-<]	4.16 (0.52)	6.96 (0.10)	11.1 (0.52)	1170 ( 31.9)	373
2. *decade-complement*		[-=]	9.65 (1.56)	10.00 (0.00)	19.7 (1.56)	1187 ( 75.2)	115
3. *decade-supercomplement*		[->]	5.23 (0.67)	12.97 (0.10)	18.2 (0.68)	2224 ( 76.8)	327
(b) non-round augend ( u>0 )^a^							
4. *subcomplement*		[<<]	26.84 (0.78)	5.45 (0.09)	32.3 (0.79)	2881 ( 78.1)	283
5. *covert complement*		[<=]	33.06 (1.70)	7.14 (0.26)	40.2 (1.76)	2852 (133.8)	51
6. *overt complement & pair:*							
a*. direct complement*		[=-]	27.94 (1.06)	10.52 (0.31)	38.5 (1.15)	2650 (117.1)	145
b*. indirect complement*		[=-]	26.48 (0.96)	9.55 (0.28)	36.0 (0.96)	2846 (111.9)	168
c*. pair*		[=−]	24.12 (0.77)	10.00 (0.00)	34.1 (0.77)	2732 (104.4)	298
d*. direct complement-pair*		[=-]	24.58 (1.10)	10.00 (0.00)	34.6 (1.10)	2072 (106.9)	121
e*. indirect complement-pair*		[=-]	24.36 (1.11)	10.00 (0.00)	34.4 (1.11)	2293 (135.8)	112
7. *supercomplement*		[<>]	27.02 (0.50)	10.10 (0.10)	37.1 (0.50)	4169 ( 65.5)	762
8. *covert complement 2*		[>=]	25.46 (1.49)	12.92 (0.20)	38.4 (1.53)	4293 (228.5)	74
9. *supercomplement 2*		[>>]	22.42 (0.77)	14.45 (0.09)	36.9 (0.79)	5467 (123.9)	284

^a^ This typology is based on the addition types for adding two addends, as defined in table 4.

^b^ Excluding 45 outliers (1.42% of 3158 pairwise additions, exceeding the overall mean by more than 3 s.d.).

#### (ii) Visualizations

The following visualization further illustrates the results of study 2:

— figure 9:
  the individual panels show the mean addition latency as a function of *augend unit* (*u*) or *addend size* ($a_{1}$ or $a_{2}$). Again, the colour gradient illustrates that addition latency corresponds closely to the pattern of qualitative *addition types* for any two of three candidate elements of an addition.
